# Supplementary material for: Are carbon emissions trading and green financial instruments synergistic? -Comprehensive quantitative research based on content analysis
Source: PLoS One. 2024 Mar 7;19(3):e0298601. doi: 10.1371/journal.pone.0298601 (PMC10919601; doi:10.1371/journal.pone.0298601)
Supplement: S1 Data — (ZIP) [file pone.0298601.s001.zip › Data/地级市Z_F工作报告.zip!/║╙─╧/╚2├┼╧┐╩╨/└┤╫╘╣1⁄2╓┌║┼í╛┬φ┐╦╩2╛▌═°í┐_2016.docx]

各位代表：

　　现在，我代表市人民政府向大会报告工作，请予审议，并请各位政协委员和其他列席人士提出意见。

　　一、“十二五”主要成就及“十三五”目标任务

　　“十二五”以来，宏观经济错综复杂，改革发展面临前所未有的多重困难和严峻挑战。在省委、省政府和市委的坚强领导下，全市上下全面落实党的十八大和十八届三中、四中、五中全会精神，深入贯彻习近平总书记系列重要讲话精神，抢抓国家“一带一路”战略、河南省“三大国家战略规划”实施等机遇，积极适应经济新常态，主动作为，敢于创新，善抓落实，交出了一份经济平稳增长、发展方式加快转变、群众生活持续改善、综合实力再上台阶的合格答卷。

　　综合实力竞争力显著提升。经济总量不断壮大，“十二五”期间，全市生产总值年均增长9.3%，人均地区生产总值5.6万元，均为2010年的1.4倍。规上工业增加值年均增长10.7%，一般公共预算收入年均增长13.6%，全社会固定资产投资年均增长21.3%，社会消费品零售总额年均增长14.4%，进出口总值年均增长21.8%。城乡面貌焕然一新，商务中心区、特色商业区建设成效初显，沿黄半岛景观带建设、旧城区和城中村改造扎实推进，“城市管理年”和“洁城行动”深入开展，中心城区品位魅力明显提升，县（市）城区带动力持续增强，全市城镇化率达到51.3%，农村人居环境显著改善，21个村入选全国和全省“美丽乡村”试点。

　　发展方式加快转变。三次产业结构由2010年的8.0∶68.5∶23.5转变为9.4∶60.1∶30.5。工业经济提质发展，黄金、铝等主导产业占规上工业增加值的80%以上，高新技术产业占规上工业增加值的比重提升12.3个百分点，荣获国家科技进步先进市称号。现代服务业提速发展，在全省率先建设商务中心区，服务业增加值年均增长9%，占生产总值比重比2010年提高7个百分点。现代农业加快发展，特色农业产值占比85%以上。万元GDP能耗、万元工业增加值能耗分别累计下降17%和30%，化学需氧量、氮氧化物、二氧化硫、氨氮排放分别削减9.9%、15.5%、10.5%和13.4%，森林覆盖率达50.7%，保持全省第一，获得国家园林城市、国家森林城市等称号。

　　发展支撑日益增强。郑西高铁、三淅高速、连霍高速改扩建等建成通车，“三纵四横”交通网络不断完善。三门峡海关等三大通关机构顺利建成，国家果品及果蔬汁检测重点实验室落户。与省工、农、中、建、农发5家银行开展战略合作，落实协议资金1236亿元。“十二五”期间共实施重点项目1269个，完成投资3670亿元，中金黄金产业园等一批重大项目落地。河南科技大学应用工程学院挂牌成立，填补了我市本科院校的空白。崤函古道石壕段遗址联合申遗成功，实现入选世界文化遗产“零”突破，为转型发展提供了有力支撑。

　　改革开放扎实推进。重要领域和关键环节改革持续深化，非行政许可审批事项全面取消，医药卫生、财税金融、工商登记等领域改革扎实推进。积极开展招商引资，累计签约项目745个，总投资5280亿元，实际利用外资年均增长20.4%，实际到位省外资金年均增长23.4%。晋陕豫黄河金三角区域合作上升为国家战略。

　　群众生活不断改善。持续实施省市“十项重点民生工程”，财政民生支出累计520.7亿元，占财政支出的70%，是“十一五”的2.4倍。城镇累计新增就业27.9万人，转移农村劳动力40万人，城乡居民人均可支配收入分别年均增长10.1%和13.6%，农民人均纯收入增速连年位居全省前列。扎实推进新一轮脱贫攻坚，实现13.5万人稳定脱贫。社会大局和谐稳定，在全省率先荣获全国社会治安综合治理最高荣誉“长安杯”。

　　各位代表！“十二五”时期的辉煌成就，集中展现了全市人民的伟大创造力，极大增强了全市上下的自信心和凝聚力，必将激励我们在实现全面建成小康社会新征程上奋力前行！这些成绩的取得，是省委、省政府和市委的正确领导的结果，是市人大、市政协大力支持的结果，是全市人民团结拼搏的结果。在此，我代表市人民政府，向全市各行各业的建设者，向各位人大代表、政协委员、各民主党派，向驻峡解放军、武警官兵、公安民警，向广大客商以及关心支持三门峡发展的各界人士，致以崇高的敬意和衷心的感谢！

　　五年奋斗，五年发展，我们深深地感到，要加快转型步伐，实现华丽转身，必须始终坚持发展第一要务，在发展中调整，在调整中提升；必须主动适应经济新常态，不断以改革创新的举措激发活力、转换动力；必须坚持以人为本，始终把民之所望作为工作所向，多谋民生之利，多解民生之忧；必须加强作风建设，以精神状态的上行动力应对经济运行的下行压力，埋头苦干、久久为功。这是全市人民辛劳和智慧的结晶，我们一定要倍加珍惜、牢牢坚持，使之成为赢得未来发展的制胜法宝！

　　各位代表！往事可鉴，来者可追。“十三五”时期是全面建成小康社会的决胜阶段，也是我市加快转型发展的关键时期，我们一定要准确把握我市发展所处的历史阶段和经济新常态带来的趋势变化，以舍我其谁的勇气，奋力开拓发展的新境界！

　　根据市委意见，市政府编制了《三门峡市国民经济和社会发展第十三个五年规划纲要（草案）》，已与纲要说明一起印发会议，提请审议。

　　“十三五”全市经济社会发展的指导思想是：深入贯彻落实党的十八大和十八届三中、四中、五中全会精神，主动适应国际经济新形势新变化和国内经济新常态，以“四个全面”战略布局为总引领，全面贯彻创新、协调、绿色、开放、共享五大发展理念，紧紧围绕“一带一路”重要节点城市、黄河金三角区域中心城市、转型发展示范城市、休闲文化城市四大战略定位，不断优化生产、生活、生态三大空间布局，着力实现产业转型升级和中心城区转型两大突破，争取提前一年实现全面建成小康社会奋斗目标。

　　主要预期目标是：在提高发展质量和效益基础上，生产总值增速和主要人均指标高于全省平均水平，到2019年生产总值和城乡居民人均可支配收入比2010年翻一番，人民生活水平普遍提高，生态环境更加优良，转型升级实现突破，先进制造业、现代服务业、战略性新兴产业比重大幅提升，动能转换基本完成，城市带动能力显著增强，城乡发展日益协调，初步形成优势产业强大、新兴产业壮大、城市规模扩大的区域中心城市，跨入全省综合实力强市行列，现代化建设迈出坚实步伐，黄河明珠、天鹅之城展现出更加美好的发展前景。

　　实现上述目标，必须深入落实“54321”转型发展战略：

　　一是贯彻五大发展理念。把创新发展作为关键动力，强化理念、方法、路径创新，培育转型发展新动能。把协调发展作为有力保障，统筹城乡、区域、经济社会协调发展，增强发展的整体效能。把绿色发展作为内在要求，促进产业发展生态化、生态建设产业化，形成人与自然和谐发展新格局。把开放发展作为必由之路，扩大开放招商，深化区域合作，打造内陆开放新高地。把共享发展作为根本目的，推进脱贫攻坚，增进民生福祉，创造幸福和谐新生活。

　　二是明晰四大战略定位。抢抓国家实施“一带一路”战略机遇，努力建设“一带一路”重要节点城市。充分发挥比较优势，加快建设区域交通、物流、商贸、旅游中心，打造黄河金三角区域中心城市。坚持做强工业、做大服务业、做优农业，加快构建现代产业体系和自主创新体系，打造转型发展示范城市。依托生态环境优势和历史文化优势，加快城市转型发展，提升城市气质，增添城市魅力，打造休闲文化城市。

　　三是优化三大空间布局。优化生产空间布局，以产业集聚区、商务中心区、特色商业区为载体，推动形成产业升级与城市提升双轮驱动、互相促进的发展格局。优化生活空间布局，着力构建“一核两翼三带多点”的新型城镇体系，建设美丽乡村，推动城乡基本公共服务均等化，形成城乡互促联动、共同繁荣新局面。优化生态空间布局，科学划定绿色系统红线、生态保护红线和永久基本农田红线，加强生态文明建设，让美丽与发展同行。

　　四是着力实现两大突破。努力实现产业转型升级突破，建设新型工业化城市、高成长服务业先进市、特色农业强市、网络经济大市。努力实现中心城区转型突破，拉大城市框架，强化产业支撑，完善城市功能，提升文化品位，增强城市影响力和辐射带动力。

　　五是提前实现一大目标。坚持发展第一要务，持续保障改善民生，到2019年实现生产总值、城乡居民收入比2010年翻一番，提前一年实现全面建成小康社会目标。

　　各位代表！展望未来，奋进的目标召唤着我们，人民的期待鞭策着我们，光明的前景激励着我们，我们信心百倍。我们坚信，只要我们和衷共济、砥砺前行，我市“十三五”发展的宏伟蓝图一定能够如期实现！

　　二、2015年工作回顾

　　2015年是“十二五”的收官之年，也是我市经济社会发展极不平凡的一年。我们认真贯彻省委、省政府和市委决策部署，深入开展“三严三实”专题教育和“我为三门峡发展作贡献”主题实践，着力稳增长、保态势、抓落实，全市经济社会平稳健康发展。全市生产总值完成1261亿元，增长3.5%。一般公共预算收入93.9亿元，增长1.6%。规上工业增加值602亿元，增长1.8%。全社会固定资产投资1551亿元，增长15.9%。社会消费品零售总额397亿元，增长11.6%。进出口总值4.4亿美元，增长84.9%。城乡居民人均可支配收入分别达到2.38万元和1.1万元，实际增长5.4%和8.4%。

　　（一）重大项目建设取得新突破。全市23个省重点项目完成投资201亿元，占年度目标的140.5%。245个市重点项目完成投资858亿元，占年度目标的142%，其中亿元以上工业转型升级项目54个，完成投资295亿元。中金黄金产业园一期、连霍高速改扩建等48个项目建成，蒙华铁路等200多个重大项目加快推进。积极争取国家专项建设基金，54个项目争取基金39亿元，位居全省第三位，可带动全社会固定资产投资711亿元。12个项目纳入国家、省PPP示范项目。科学编制“十三五”规划，谋划重大项目1546个，总投资1.18万亿元。

　　（二）产业转型升级取得新进展。工业经济转中趋稳，积极培育装备制造、新材料、新能源、生物医药等战略性新兴产业，河南能化汽车零部件产业园、乐氏同仁药业中药产业园等项目加快推进。新认定国家级高新技术企业7家、院士工作站等各类创新平台15家。三门峡产业集聚区创业服务中心被认定为国家级孵化器。高新技术产业完成增加值104亿元，占规上工业增加值的17.2%。特色农业加快发展，粮食总产7.2亿公斤，占目标任务的143%。果品总产23亿公斤，其中苹果18.6亿公斤。收购烟叶57.5万担，规模和质量继续保持全省首位。食用菌突破1.76亿袋。省、市农业产业化集群达到39个，农业龙头企业206家，农民专业合作社1100家。“三品一标”农产品数量133个，“灵宝苹果”品牌价值171亿元，跻身全国初级农产品类地理标志产品十强。建成全省首家农产品质量安全追溯服务中心，被确定为全国信息化基地建设整体推进市。现代服务业扩量提质，第三产业占GDP的比重比上年提高3.3个百分点。现代物流业加快发展，万石物流等项目进展顺利。义马市农商行正式挂牌，金融对经济增长的贡献率达6.8%。黄河公园等4家景区成功创建4A级景区。成功举办第21届黄河文化旅游节、白天鹅旅游季等大型活动，全年接待国内外游客2687万人次，增长13.2%，旅游总收入225.6亿元，增长11.3%。

　　（三）城乡建设取得新提升。陕州区正式挂牌，三门峡进入“一城两区”时代。商务中心区完成投资75亿元，基础设施建设基本完成，2000户居民实现回迁，3所配套学校正在建设，50多个招商项目有序推进，昔日的荒山荒沟，正在成为“生态宜居、产城融合”的城市新中心。虢国中路等4条新建、改建道路通车，新增供热面积30万平方米。上村一期等旧城区和城中村改造项目进展顺利。实施“魅力天鹅城·美丽三门峡”洁城行动，施行“以克论净·深度清洁”，城区路面达到“双十”标准。休闲文化城市建设扎实推进。义马、渑池基础设施对接加快，灵宝市被列入省级城镇化综合试点城市，卢氏县城跨洛河拓展，县（市）城区面貌持续改观。新增美丽乡村示范村61个，总数达138个。扎实推进农村环境综合整治，76%的行政村达到“三无一规范一眼净”，走在全省前列。农村基础设施不断改善，大石涧水库项目设计通过国家评审，国家抗旱规划项目等一批重大水利工程全面实施。

　　（四）发展活力取得新增强。各项改革深入推进，政府权力清单全面建立，减少行政审批事项26项。积极推行工商税务“三证合一”登记制度，新增市场主体9868家，增长13.2%。交通运输行政执法体制改革全面完成，党政机关公车改革、机关事业单位养老保险制度改革和农村土地确权颁证扎实推进。开放合作不断扩大，积极争取省政府出台《河南省晋陕豫黄河金三角区域合作规划实施方案》。推行“三专一委托”招商工作法，签约招商项目176个，总投资730.7亿元，履约率85.8%，开工率67.6%，资金到位率31.3%。引进实施中电投等央企合作项目11个，完成投资145亿元。对外贸易完成出口1.75亿美元，增长47.3%。对外承包工程和劳务合作营业额增长59.1%。4家公司在“新三板”挂牌，8家公司在上海股权托管交易中心挂牌，27家公司进入省上市“后备队”。发展载体日趋完善，推进产业集聚区“三上一提高”，三门峡产业集聚区在全省观摩中取得小组第一，渑池县产业集聚区进入全省“十快”。全市产业集聚区规上工业主营业务收入1877亿元，成为带动区域经济发展新的增长极。服务业“两区”建成区面积达到4.6平方公里，入驻企业941个，从业人员4.3万人。高阳山温泉国际度假区等11个项目建成投用，渑池县特色商业区被评为全省“十快”。电子商务蓬勃发展，阿里巴巴·三门峡产业带运营平稳，培育省、市电商示范企业27家。成立市特色农产品电子商务中心，农业电子商务千县万村计划和阿里巴巴“淘宝村”项目加快推进，卢氏县被确定为全国电子商务进农村综合示范县。全市电商交易额15亿元，网络零售额4亿元，均实现历史性突破。

　　（五）群众生活取得新改善。加大财政民生投入，积极实施省、市“十项重点民生工程”。努力扩大就业，扎实推进大众创业、万众创新，市创业孵化基地挂牌运营，完成全民技能振兴工程培训9.5万人次。持续实施“崤函创业扶持行动”，发放创业担保贷款6.1亿元，扶持创业7702人。城镇新增就业6.1万人，城镇登记失业率2.8%。加强社会保障，社会保险制度体系不断完善，城乡低保等覆盖面及标准位居全省前列。新开工保障性住房4624套，基本建成2425套。加大扶贫开发力度，3.7万农村贫困人口实现稳定脱贫，解决14.9万农村居民和在校师生饮水安全问题。积极发展社会事业，河南科技大学应用工程学院首批本科专业招生，学前教育行动计划二期等教育工程深入实施。县级公立医院改革扎实推进，市中心医院晋升“三级甲等医院”，单独二孩政策稳妥实施，免费孕前健康检查实现城乡全覆盖，人口自然增长率4.25‰。文化体制改革扎实推进，义马市荣获“全国文化先进县”称号，函谷关文化产业园入选省级文化产业示范园区，“政府采购百场戏”“舞台艺术送农民”扎实开展。群众健身活动蓬勃开展。新行政服务中心投入使用。加强精神文明建设，社会主义核心价值观和道德建设宣传教育工作在全省受到表彰，“文明河南·暖暖新年”系列活动成效显著，志愿服务形成了“爱心之家”“扶人协会”两大品牌，新增全国文明村镇2家，涌现出一批省级精神文明先进个人。加强生态文明建设，大力实施“蓝天碧水”工程，完成省政府下达的污染治理目标任务，万元GDP能耗、万元工业增加值能耗分别下降0.7%和2.1%。大力发展清洁能源，风电装机规模34万千瓦，占全省一半以上。加强林业生态建设，新造林42.3万亩，森林抚育和改造34.5万亩。创新社会治理，预防和化解社会矛盾，全面排查处置非法集资风险，信访形势总体稳定。深入开展“三查三保”活动，安全生产和食品安全形势平稳，社会大局和谐稳定。国防动员和双拥共建深入开展，军政军民团结更加巩固。统计、审计、外侨、民族、宗教、对台、史志、人防、防震减灾、无线电、黄河河务、妇女儿童、残疾人、老龄、慈善等工作取得新成绩。

　　各位代表！在肯定成绩的同时，我们也清醒地看到，我市经济社会发展中还存在着一些突出矛盾和问题：主导产业产品以原材料和初加工为主，产业层次低，产品附加值不高，距离中高端水平还有较大差距。接替产业规模偏小，新兴产业培育不足，对经济社会发展的支撑作用有限。财政收入增长放缓，公共服务水平不高，脱贫攻坚任务繁重，民生领域欠账较多等。特别是在当前经济下行压力加大的形势下，一些企业生产经营困难，有的行业困难加剧，问题更加凸显。同时，法治政府建设仍需加强，一些地方和部门服务意识不强，行政效能不高，消极腐败现象依然存在。对此，我们一定高度重视，认真解决，尽心竭力做好政府工作，决不辜负人民重托！

　　三、2016年重点工作

　　今年是实施“十三五”规划、全面建成小康社会决胜阶段的第一年，也是推进结构性改革的攻坚之年。做好政府工作，必须全面贯彻党的十八大，十八届三中、四中、五中全会和中央、省委、市委经济工作会议精神，认真落实市委“54321”转型发展战略，以稳中求进、转型发展为主基调，牢牢把握产业升级和城市转型两大突破，着力稳定经济增长，着力培育优势产业，着力抓好城市建设，着力深化改革开放，着力推进脱贫攻坚，着力保障改善民生，促进经济社会持续平稳健康发展，不断提升区域核心竞争力，确保“十三五”良好开局。

　　经济社会发展主要预期目标是：地区生产总值增长8%以上，一般公共预算收入增长7%以上，规模以上工业增加值增长9%，固定资产投资增长15%以上，社会消费品零售总额增长12%，进出口总值增长10%，城镇居民人均可支配收入实际增长9%以上，农村居民人均可支配收入实际增长10%以上，节能减排、环保等约束性指标完成省定目标。

　　综合分析各方面情况，今年我们发展面临的困难较大、挑战较多，但困难和挑战并不可怕，我们的发展从来都是在应对困难和挑战中前进的，只要我们全面落实省委、省政府和市委各项决策部署，发挥好政策叠加的机遇优势，利用好作风建设的丰硕成果，以壮士断腕的决心推进转型发展，以舍我其谁的勇气建设省际中心城市，以敢于担当的责任感推动脱贫攻坚，以驰而不息的精神推动作风转变，万众一心、共克时艰，就一定能够披荆斩棘、破浪前行，赢得“十三五”发展的开门红！

　　今年要重点做好六个方面的工作：

　　（一）稳定经济增长，确保经济运行在合理区间。把稳增长、保态势作为全局工作的突出任务，全力抓好经济运行，推动全市经济发展提质增速。

　　加快项目建设。发挥投资对稳增长的关键作用，抓好22个省重点项目，年度完成投资152.9亿元。全力推进242个市重点项目，年度完成投资550亿元。加快实施已获得国家专项基金的54个项目。持续做好国家专项建设基金争取工作，力争继续在全省保持领先位次。谋划筛选适宜PPP模式的新项目，争取纳入国家、省PPP项目推介库。

　　加强企业服务。坚持分类指导、一企一策，加大对规上工业企业、限额以上商贸企业、农业龙头企业、小微企业服务力度，搞好产销、银企、用工、产学研“四项对接”，帮助解决实际困难。稳妥有序处置“僵尸企业”，对产品有市场、有效益但暂时遇到困难的企业，积极争取中央、省专项奖补支持。规范清理涉企收费，帮助企业降低生产成本和税费负担，为企业破解发展瓶颈提供“保姆式”服务。加强企业家队伍建设，推动提升经营理念和管理水平。

　　强化要素保障。强化土地、能源、资金等要素支撑，抓好资源整合开发，加快推进渑池煤下铝等国家整装勘查项目。实行有保有控的供地政策，优先保障重点项目用地。积极拓宽大用户直供、大工业用电渠道，做好煤电油运等要素供给。加强政银企合作对接，完善政保贷、助保贷、过桥资金池等政策措施，帮助企业解决融资难题。加强与国开行、农发行等金融部门对接，做好国家专项建设基金项目的融资工作。设立5000万元经济转型发展专项资金，研究建立推动产业升级和城市转型的若干基金，重点支持对全市经济发展贡献大的工业企业、商贸企业、电商企业和金融机构，确保优势产业和新兴产业今后发展的资金需求。

　　（二）坚持发挥优势顺应趋势，加快推动产业升级突破。坚持把培育优势产业、新兴产业作为转型发展的主攻方向，推动产业规模壮大、结构优化、层次提升。

　　加快建设新型工业化城市。以《中国制造2025河南行动纲要》和省委、省政府“十大重点产业”为指导，改造提升传统工业，培育壮大战略性新兴产业，强化工业化信息化深度融合，推动工业提质增效。黄金工业，抓好中金集团黄金产业园、灵宝4万吨锂电铜箔、志成金铅技改提升等项目，拉长黄金、铜精深加工产业链，建设全国重要的黄金珠宝加工交易基地和铜材生产加工基地。铝工业，抓好戴卡轮毂汽车零部件产业园、同人铝业年产60万吨铝合金铸造及深加工、锦江集团汽车轻量化等项目，推动铝工业向高端产品加工转变。煤化工，抓好开祥化工产品结构调整、义煤综能公司甲醇蛋白等项目，推进煤化工产业向精深加工发展。战略性新兴产业，抓好义腾新能源年产3000万套锂离子电芯、康耀电子年产300万片导电玻璃、乐氏同仁药业中药产业园等项目，加快装备制造、新材料、电子信息、生物医药等产业发展。突出创新驱动，支持行业骨干企业重大技术攻关、智能化改造和重大产业基地建设，推动电动汽车、新材料、新能源等重点行业领域实现技术突破。加快高新技术产业和创新平台建设，争取高新技术企业总数达到45家，高新领域企业总数突破150家，新认定市级以上创新平台10家。

　　加快建设高成长服务业先进市。以物流、金融等专业化服务为引领，加快发展生产性服务业，以休闲、健康等个性化消费为导向，加快发展生活性服务业，充分发挥服务业对转型发展的带动作用。提速发展现代物流业，确保锦江集团“一带一路”大宗商品交易中心等标志性项目开工建设，加强城乡物流配送体系建设，打造黄河金三角区域物流中心。积极发展金融服务业，陕州区农商行年内挂牌开业，灵宝市农信社达到农商行组建标准。加快组建村镇银行，实现县（市）村镇银行全覆盖。积极引进域外金融机构在我市设立分支网点。积极稳妥发展互联网金融、绿色金融、金融租赁等新兴业态。壮大提升旅游业，迎接正在兴起的大众旅游时代，整合自然山水和历史文化等旅游资源，加快发展全域旅游。推进旅游综合服务中心、陕州地坑院文化旅游区等重点项目，年内完成投资16亿元，创建4A级景区1家、省级旅游度假区1家。办好第22届黄河文化旅游节暨投资贸易洽谈会。积极发展大健康产业，依托“青山绿水好空气”生态环境优势，大力发展休闲、健身、养老、疗养等大健康产业。积极促进批发零售、住宿餐饮等传统服务业提档升级，推动教育、医疗、文化、体育等服务业加快发展。稳步发展房地产业，落实省、市政策措施，促进新市民在城镇购房，鼓励农民进城购房，争取房地产销售增长10%以上。建立购租并举住房制度，提高棚改货币化安置率。

　　加快建设特色农业强市。发挥特色农业优势，加强农业供给侧结构性改革，打造高品质、致力高品级、形成大品牌，推动农业向质量效益、生态化、品牌化转变。种植农业坚持生态化、品级化、有机化，大力推进地理标志农产品、无公害农产品和绿色食品等标志认证，加强生态原产地产品保护，使农产品真正成为大自然馈赠给人类的“精灵”。加工农业坚持链条化、效益化、品牌化，瞄准市场需求，加快发展果汁、食用菌、畜禽、核桃等加工产业，提高农产品效益。充分发挥农业龙头企业的引领带动作用，高起点嫁接利用汇源果汁、雏鹰农牧等知名品牌，实现跨越发展。农业产业集群坚持规模化、融合化、循环化，积极引进农业龙头企业，大力发展家庭农场、专业大户、农民专业合作社、股份合作经济组织等新型农业经营主体，持续提升果品、畜牧等集群发展水平。推进农村一、二、三产业融合发展，促进观光农业、加工农业、乡村旅游有机融合，形成全链条、全体系、全循环的现代农业产业发展体系。强化农业基础支撑，开工建设大石涧水库等水利工程，加快灌区建设和节水改造，继续实施中小河流治理和山洪、地质灾害防治。

　　加快建设网络经济大市。把网络经济作为创新发展、跨越发展的切入点和突破口，不断强化基础支撑、平台支撑和人才支撑，推动以电子商务为先导的网络经济加速发展。筹办好首届“互联网+”联盟峰会，搭建面向中西部的网络经济发展平台。加快电商园区建设，推进商务中心区电商大厦、颐高集团电商产业园等项目，谋划打造三门峡快递物流园，抓好市电子商务创业园、市众创空间暨创业孵化基地等各类园区运营发展。抓好产业带运营，依托阿里巴巴·三门峡产业带运营优势，大力发展平台经济，扶持本土电商企业做大做强，谋划举办三门峡网上购物节，积极发展跨境电子商务。壮大农业电子商务，围绕“一村培育一品、一品叫响一店、一店带富一片”的目标，重点推进卢氏县、灵宝市“农村淘宝”项目，引领和带动全市农业电商发展，力争70%的行政村达到“一村一品”，网络销售额超过20亿元。加强人才培育，与国内知名电商培训机构和重点院校合作，谋划推动电子商务培训机构、专业学科建设，全年培训不少于1万人次。认真办好全省农业电子商务现场会。

　　（三）突出中心城区带动，加快推动城市转型突破。把中心城区转型作为转型发展的主战场，深入落实中央、省委城市工作会议精神，突出特色，传承文脉，增添魅力，加快构建“一核两翼三带多点”城镇化格局。

　　强化业态支撑。依托产业集聚区、开发区、湖滨工业园区等载体，大力发展先进制造业。依托城郊家庭农场，大力发展都市生态农业，促进特色农业与休闲度假等充分融合。以沿黄半岛景观带为主体框架，整合旅游资源，打造黄河天鹅湖湿地旅游度假区，加快青龙涧河景观带改造提升，做大做强主城区旅游。坚持一手抓建设，一手抓繁荣，加快服务业“两区”建设，持续抓好基础设施建设和项目招商，进一步提升承载能力，培育发展金融保险、研发设计、信息服务等生产性服务业和现代商贸、娱乐健身等生活性服务业。

　　完善城市功能。强力推进商务中心区建设，确保明珠大厦等在建项目尽快竣工、武警指挥中心等项目尽快开工，加快建设学校、医院等配套设施，进一步提升承载能力，打造连接湖滨区和陕州区的城市新中心。推动中心城区基础设施扩容提升，加快综合管廊、海绵城市规划建设。打通六峰南路等7条“断头路”和黄金大道、中心大道，推进经三路等3条雨污水管网和老旧道路、桥梁改造。加快推进上村片区、崖底村等旧城区和城中村改造项目，依法处置违法违规建设行为。完善城市交通网络，争取国道310线南移项目开工建设，抓好三淅高速豫晋省界至灵宝段、国道344线、省道313线等项目建设。推动陕州区与湖滨区融合发展，加快灵宝城区功能改造，积极向东与城乡一体化示范区对接发展。加快义马、渑池一体化进程，努力建设生态宜居新卢氏。

　　提升城市品位。坚持融入自然，依托沿黄生态带、崤山—小浪底生态带、伏牛山—熊耳山生态带，打造城市生态走廊。优化城市绿地布局，推动立体绿化规划建设。坚持融入文化，大力挖掘历史文化资源，加快推进庙底沟文化遗址公园等项目建设。大力弘扬社会主义核心价值观，积极开展“践行价值观、文明我先行”等系列活动，持续抓好精神文明创建，大力倡导“做文明人，办文明事”，加强社会诚信体系建设，持续开展好横渡母亲河、黄河大合唱、万人帐篷节等品牌活动，涵养城市底蕴和气质，塑造城市形象新名片。坚持融入现代，推进智慧城市规划建设，启动实施数字化城市管理指挥中心项目，持续开展“洁城行动”，中心城区主次干道“以克论净”达到“双五”标准，加强背街小巷的治理和管理，提升城市精细化管理水平，让我们的城市更有序、更干净、更美丽、更宜居。

　　统筹城乡协调。坚持“一基本两牵动三保障”，加快户籍制度改革和居住证制度“双落地”，维护进城落户农民土地承包权、宅基地使用权、集体收益分配权，将农民户口变动与“三权”脱钩，有序推动进城农民市民化。支持函谷关镇、洪阳镇、观音堂镇、官道口镇等重点镇因地制宜发展主导产业，提升城镇发展水平。巩固提升138个美丽乡村示范村，新启动62个示范村创建，打造6条示范带。持续开展农村环境综合整治，实施农村生活垃圾治理5年专项行动，力争95%以上行政村基本达到“三无一规范一眼净”，40%以上行政村达到改善农村人居环境达标村标准。

　　（四）深化改革开放，增添经济社会发展动力。坚持以改革促发展，在开放中扩内需，激发转型发展动力活力。

　　持续深化改革。加快财税体制改革，积极推进预决算公开，加强政府债务管理。加快经济发达试点镇改革，推进灵宝市豫灵镇等4个试点镇成为县域经济新的增长点。深化投融资体制改革，加快实施PPP项目。全面完成土地确权登记颁证工作，稳妥推进农村各项改革，激发农村发展活力。加快医药卫生体制改革，稳步推进县级公立医院等其他领域改革。

　　加大招商引资。围绕培育优势产业，加强招商引资顶层设计，依托我市黄金及有色金属、铝工业等优势产业和黄金珠宝加工基地等重点项目，积极招大引强。用好“三专一委托”等工作方法，坚持产业链招商、驻地招商、以商招商，把握企业“抱团转移”新趋势，加强与行业协会、企业商会、中介机构对接合作，进行整体招商，着力提高招商引资实效。完善招商责任推进机制，加强签约项目的落地服务，提高签约项目履约率、开工率、资金到位率。办好“百名客商三门峡行”活动，争取第22届黄河文化旅游节暨投洽会、第四届特博会成功签约一批好项目。

　　深化开放合作。抓好外经外贸，充分发挥通关优势，主动融入“一带一路”，抓好省级苹果及果汁出口基地建设，培育食用菌等出口龙头企业，新增进出口备案企业50家以上。鼓励煤炭、黄金、水利工程等优势企业“走出去”，到境外承包工程、建立生产基地。强化区域合作，落实《晋陕豫黄河金三角区域合作规划》，加强区域内基础设施对接，加快蒙华铁路、黄河公铁两用大桥等项目建设。

　　（五）坚持绿色发展，建设美丽宜居新家园。坚持治、防、管一体抓，持续实施蓝天、碧水、乡村清洁工程和城市河流清洁行动计划，打赢污染治理攻坚战，努力让山更青、水更绿、空气更清新，环境更舒适更宜居，叫响“黄河三门峡，美丽天鹅城”品牌。

　　强力推进污染减排。全面落实《河南省减少污染物排放条例》，完成省政府下达的减排任务。加大工业污染治理力度，坚决淘汰落后产能。继续严把项目建设审批关，落实“四个不批”，规范建设项目竣工环保验收，从源头上杜绝污染。加强污水处理厂建设和运营监管，确保污水达标排放。

　　深入开展大气、水污染防治。加强大气污染治理，突出做好工业大气污染防治、机动车污染控制、扬尘综合治理、农作物秸秆禁烧、油气回收治理等工作，有效削减PM10和PM2.5浓度，遏制雾霾天气高发态势。继续深化断面水质达标行动，抓好涧河等重点流域水污染防治、卫家磨水库等饮用水源地保护、南水北调中线工程水源保护工作，守好水资源管理“三条红线”。

　　抓好生态环境建设。全面落实《河南省湿地保护条例》和《三门峡市白天鹅保护区管理办法》，提升黄河湿地生态系统整体功能，在全市形成“保护湿地、留住天鹅”的浓厚氛围。持续加强林业生态建设，完成生态造林30万亩，森林抚育和改造20万亩。完成省、市级生态村和生态乡镇创建任务。

　　（六）保障改善民生，持续增进民生福祉。继续实施好省、市“十项重点民生工程”，让人民群众更多地分享改革发展成果。

　　打好脱贫攻坚战。贯彻精准扶贫、精准脱贫基本方略，坚持“转、扶、搬、保、救”五措并举，大力实施“五个一批”工程，健全扶贫攻坚联动机制，完善定点扶贫机制和选派第一书记驻村帮扶机制，强化易地扶贫搬迁顶层设计和模式创新，积极创建政策性金融扶贫试验示范区，引导资金、土地、人才、技术等要素向贫困地区集聚，实现3.32万农村贫困人口稳定脱贫，确保脱贫攻坚首战必胜！

　　积极扩大就业。持续开展大众创业、万众创新，继续实施“崤函创业扶持行动”，全年发放创业担保贷款5亿元以上。深入推进全民技能振兴工程，不断提高就业能力，确保城镇新增就业3.6万人以上。

　　强化社会保障。全面实施全民参保登记计划，深化机关事业单位养老保险制度改革，继续提高企业退休人员基本养老金。继续完善城镇基本医疗保险制度，加快养老、医疗、失业、工伤、生育保险市级统筹。深化医疗保险付费方式改革，将职工医保、城镇居民医保政策范围内住院费用支付比例分别提高到80%、75%左右，新农合和城镇居民医保年人均补助标准再提高30元。城乡低保财政补差水平月人均分别提高15元和12元。加强保障性住房建设，货币化安置比例达到50%以上。健全社会救助工作机制，建立经济困难高龄失能老人补贴、困难残疾人生活补贴和重度残疾人护理补贴制度。

　　发展社会事业。大力提升教育治理能力，全面提高教育教学质量。扎实推进“全面改薄”和“扩充城镇义务教育资源”工作，新建、改扩建16所幼儿园和132所中小学，改善基础教育办学条件；加快普及高中阶段教育。实施乡村教师支持计划，加大农村寄宿制学校建设力度。加快推进中职教育资源整合，在商务中心区开工建设1所高标准民办学校。建立健全贫困生资助体系，免除经济困难普通高中学生学杂费，努力让所有贫困家庭的孩子都能接受公平的教育！巩固基本药物制度，保持二级公立医院基本药物使用比例不低于45%，三级公立医院不低于30%。坚持计划生育基本国策，实施全面两孩政策，加大出生缺陷干预力度，开展产前基因检测，提高新生人口素质。抓好文化重点项目建设、文艺精品创作、文化惠民等重大文化工程，编辑三门峡文化丛书，推动传统媒体、新型媒体融合发展。加快文化产业发展，完善公共文化服务体系。大力发展体育事业，广泛开展全民健身行动。

　　创新社会治理。推进平安三门峡建设，健全社会治安防控体系，推进“一村一警”工作，争创全国社会治安综合治理优秀市“四连冠”。强力推进隐患排查和打非治违专项行动，坚决遏制重特大事故发生。完善体制机制，强化食品药品安全监管，确保群众“舌尖上的安全”。健全完善矛盾纠纷排查化解制度，着重解决信访突出问题。全力抓好非法集资处置和风险防控，最大限度维护群众利益，维护社会稳定。

　　继续加强国防动员、国防教育和国防后备力量建设，支持驻三门峡解放军、武警部队、民兵、预备役和人民防空建设，争创全国双拥模范城“五连冠”。支持工会、共青团、妇联等群团组织更好发挥桥梁纽带作用，继续做好统计、外侨、对台、民族、宗教、史志、气象、防震减灾等工作。

　　四、加强政府自身建设

　　深入贯彻落实新发展理念，把全面建成小康社会使命扛在肩上，把万家忧乐放在心头，不断提升政府治理能力和公共服务水平，建设人民满意的法治政府、效能政府、廉洁政府和服务型政府，营造良好政务环境。

　　推进依法行政。认真贯彻法治政府建设实施纲要，坚持依法履职，把政府活动全面纳入法治轨道，自觉运用法治思维和法治方式推动工作，法定职责必须为，法无授权不可为。健全依法决策机制，认真履行重大决策法定程序。推进服务型行政执法，全面落实行政执法责任制，加强行政执法监督，促进严格规范公正文明执法，启动“七五”普法。依法接受人大及其常委会法律和工作监督，主动接受政协民主监督，严格执行人大及其常委会决定，认真办理人大代表建议和政协委员提案。加强政府协商民主工作，落实与民主党派、工商联、无党派人士联系制度，广泛听取意见建议，自觉接受社会监督和舆论监督，让权力始终在阳光下运行！

　　提升政府效能。坚持勤勉履职、恪尽职守，全面提高政府执行力。深化政府机构改革和事业单位分类改革，坚持“放、管、服”三管齐下，继续取消和下放行政审批事项，清理规范行政审批中介服务，全面建立权责清单制度并实行动态管理，坚决把该放的放开、放到位，该管的管住、管好，激发市场活力，增强经济发展内生动力。推行行政审批标准化，推广“互联网+政务服务”，推进政务公开，让企业和群众办事更便捷！

　　加强廉政建设。坚持纪严于法、纪在法前，严明政治纪律和政治规矩，履行主体责任和“一岗双责”，全面落实党风廉政建设责任制。加强党规政纪教育，严格执行廉洁自律准则和各项规定。深入推进公务用车制度改革，完善“三公”经费预算管理和公开制度。加强行政监察和审计监督，加大查处违法违纪案件力度，以零容忍态度惩治腐败，以反腐倡廉的实效保障发展、取信于民！

　　改进工作作风。深入践行“三严三实”，增强政治意识、大局意识、核心意识、看齐意识。认真开展“两学一做”学习教育，深化“我为三门峡发展作贡献”主题实践，督促引导各级公务人员“四问四做起”，弘扬“主动作为、敢于创新、善抓落实”的工作作风，形成“比工作、比奉献、争一流”的良好氛围，继续推进懒政怠政为官不为专项治理，驰而不息纠正“四风”。深入基层调查研究，听实话、察实情、出实招。强化教育培训和实践锻炼，提升公务人员素质能力。完善全面覆盖的督查机制，打通政策落实中的“最后一公里”。健全激励机制和容错纠错机制，为担当者担当，为改革创新者撑腰鼓劲，让广大干部愿干事、敢干事、能干成事。

　　各位代表！实干实现梦想，拼搏赢得未来。让我们更加紧密团结在以习近平同志为总书记的党中央周围，在省委、省政府和市委的坚强领导下，凝心聚力，奋发进取，为提前实现全面建成小康社会目标而努力奋斗!
